# Biomarkers of viral and bacterial infection in rhinovirus pneumonia

**DOI:** 10.3389/fped.2023.1137777

**Published:** 2023-03-15

**Authors:** Maria Hartiala, Elina Lahti, Laura Toivonen, Matti Waris, Olli Ruuskanen, Ville Peltola

**Affiliations:** ^1^ Department of Pediatrics and Adolescent Medicine, Turku University Hospital and University of Turku, Turku, Finland; ^2^ Child and Adolescent Clinic, City of Turku Welfare Division, Turku, Finland; ^3^ Department of Clinical Virology, Institute of Biomedicine, University of Turku, Turku University Hospital, Turku, Finland

**Keywords:** biomarkers, children, myxovirus resistance protein A (MxA), pneumonia, rhinovirus

## Abstract

**Background:**

Rhinovirus (RV) is often detected in children hospitalized with pneumonia, but the role of RV in causing pneumonia is still unclear.

**Methods:**

White blood cell count, C-reactive protein, procalcitonin, and myxovirus resistance protein A (MxA) levels were determined from blood samples in children (*n* = 24) hospitalized with radiologically verified pneumonia. Respiratory viruses were identified from nasal swabs by using reverse transcription polymerase chain reaction assays. Among RV-positive children, the cycle threshold value, RV subtyping by sequence analysis, and the clearance of RV by weekly nasal swabs were determined. RV-positive children with pneumonia were compared to other virus-positive children with pneumonia, and to children (*n* = 13) with RV-positive upper respiratory tract infection from a separate earlier study.

**Results:**

RV was detected in 6 children and other viruses in 10 children with pneumonia (viral co-detections excluded). All RV-positive children with pneumonia had high white blood cell counts, plasma C-reactive protein or procalcitonin levels, or alveolar changes in chest radiograph strongly indicating bacterial infection. The median cycle threshold value for RV was low (23.2) indicating a high RV load, and a rapid clearance of RV was observed in all. Blood level of viral biomarker MxA was lower among RV-positive children with pneumonia (median 100 μg/L) than among other virus-positive children with pneumonia (median 495 μg/L, *p* = 0.034) or children with RV-positive upper respiratory tract infection (median 620 μg/L, *p* = 0.011).

**Conclusions:**

Our observations suggest a true viral-bacterial coinfection in RV-positive pneumonia. Low MxA levels in RV-associated pneumonia need further studies.

## 1. Introduction

Rhinovirus (RV) is the most common cause of upper and lower respiratory tract infections in children worldwide (1–7). RV is detected in 23%–30% of children with community-acquired pneumonia (CAP) (8–11). However, the role of RV as a causative agent in CAP has been questioned, because RV is frequently detected also from children who have no symptoms at all. The need for additional diagnostic markers is obvious.

The aim of this prospective study was to investigate a viral biomarker myxovirus resistance protein A (MxA) in RV pneumonia. MxA is induced exclusively by type I (*α*, *β*) and type III (*λ*) interferons, and it is highly expressed during viral infections but not in bacterial infections (12). White blood cell count (WBC), plasma C-reactive protein (CRP), and procalcitonin (PCT) levels were studied as biomarkers of possible concomitant bacterial infection.

## 2. Methods

We enrolled children younger than 16 years of age with radiologically verified pneumonia admitted to the Department of Pediatrics and Adolescent Medicine, Turku University Hospital, from May 2014 to December 2015. We excluded children with the following severe underlying conditions: immunodeficiency, severe neurologic or developmental disability, cancer under treatment, or severe chronic cardiac or pulmonary disease other than asthma. A chest radiograph was obtained from all patients and reviewed by pediatric radiologists. Pneumonia was defined as the presence of alveolar or interstitial pneumonic infiltrates on the chest radiograph together with simultaneous signs and/or symptoms of an acute infection. Enrolled children with their caregivers were interviewed with the use of a structured questionnaire, and clinical data were collected systematically from medical records after discharge.

The Ethics Committee of the Hospital District of Southwest Finland approved the study. Before enrolment, signed informed consent was obtained from the parents of study children.

WBC, plasma CRP levels, and plasma PCT levels were determined by routine laboratory methods on admittance, in the following morning, and again if clinically needed. The concentration of MxA was determined in whole blood sample collected in the first morning after admittance. Blood was diluted 1:20 in hypotonic buffer and stored at −70°C until measurement of the MxA level by an enzyme immunoassay as described earlier (12). Nasal swab (Copan, Brescia, Italy) samples were collected on admittance, and RV-positive patients were followed weekly and nasal swabs collected to assess viral clearance. Quantitative reverse transcription polymerase chain reaction (RT-PCR) for RV, enteroviruses, and respiratory syncytial virus (RSV) was performed as previously described (13). Multiplex RT-PCR (Seegene, Seoul, Korea) was used for the detection of 16 respiratory viruses (adenovirus, influenza A and B viruses, RSV A and B, human metapneumovirus, parainfluenza virus types 1, 2, 3, and 4, RV, coronaviruses 229E, NL63, and OC43, enteroviruses, and human bocavirus) according to the manufacturer's protocol. Sequence analysis was used for RV subtyping as described elsewhere (13). The cycle threshold (Ct) values of RV were determined. *Streptococcus pneumoniae*, *Haemophilus influenzae*, *Moraxella catarrhalis*, and *Staphylococcus aureus* were cultured from nasopharyngeal swab (Copan) samples. Bacterial blood culture, *S. pneumoniae* antigen test from urine, and serum *Mycoplasma pneumoniae* antibodies were also studied.

To evaluate the role of MxA, we added a comparison group of children with a RV-positive upper respiratory tract infection (URTI) from the study by Toivonen et al. (12). From that study population, we included all RV-positive children who had respiratory symptoms, no other viral findings, and no signs of bacterial co-infection [CRP <20 mg/L and normal WBC count (5.0–15.0 × 10^9^/L)], resulting in a comparison group of 13 children.

Descriptive statistics are given as proportions, or medians with ranges or interquartile ranges (IQR). Children with RV-positive pneumonia were separately compared to children with pneumonia positive for another respiratory virus, excluding a child positive for RV and another virus, and to the comparison group of children with RV-positive URTI. For continuous data, comparisons between two groups were performed by use of the *t* test for data with equal variances assumed or not assumed according to the Levene's test. For categorical data, comparisons were performed by use of Fisher's exact test. The significance level was *p* < 0.05. Analyses were performed using SPSS version 23.0 (IBM SPSS Statistics, IBM Corp., Armonk, NY, USA).

## 3. Results

We offered study participation to parents of 39 consecutive eligible children, and 24 were enrolled (12 females and 12 males). The median age of study children with pneumonia was 3.3 years (IQR 2.3–6.6 years) and the median age of the comparison group consisting of 13 children with RV-positive URTI was 0.8 years.

Seventeen of 24 children (71%) were positive for a respiratory virus; 7 of 24 (29%) children were positive for RV and 10 of 24 (42%) were positive for a respiratory virus other than RV (RSV, *n* = 3; human metapneumovirus, *n* = 2; adenovirus, *n* = 1; coronavirus OC43, *n* = 1; human bocavirus, *n* = 1; enterovirus, *n* = 1; and influenza A virus, *n* = 1). Six RVs were typed by sequencing; 4 were of RV-A and 2 of RV-C species (Table 1). One RV-positive child had adenovirus co-infection. The median duration of symptoms before diagnosis of RV-positive pneumonia was 2.5 days. In the follow-up of children with RV pneumonia, 4 of 7 were negative for RV one week after the first sample, and all were negative for RV after two weeks. These observations showed the acute nature of RV infection in children with pneumonia.

**Table 1:** Clinical and laboratory features of 7 children with community-acquired pneumonia positive for rhinovirus.

	1	2	3	4	5	6	7
Gender	Female	Female	Male	Male	Male	Male	Male
Age, year	6.1	1.8	5.8	3.1	1.0	1.5	1.1
Rhinovirus type	C18	A63	C23	Not done	A22	A56	A58
Cycle threshold (Ct) value of rhinovirus	19.8	22.7	21.1	36.9	26.9	23.6	21.1
Other viral findings	Adenovirus						
MxA, µg/L	Not done	450	70	100	100	100	140
Highest WBC count, ×10^9^/L (normal range 5–15 × 10^9^/L)	24.5	14.4	10.3	18.9	17.8	18.0	25.7
Band forms, % of total neutrophils (normal range < 5%)	Not done	2	9	40	4	26	Not done
Highest CRP, mg/L (normal range < 10 mg/L)	190	332	345	223	290	244	298
Highest PCT, µg/L (normal range < 0.5 µg/L)	Not done	7.19	13.67	6.03	1.99	25.73	5.65
Bacterial blood culture	Negative	Negative	*S. pneumoniae*	Negative	Negative	Negative	Negative
Bacterial culture from nasopharyngeal swab	*M. catarrhalis*	*S. pneumoniae* *M. catarrhalis*	*S. pneumoniae*	Negative	Negative	*S. pneumoniae* *M. catarrhalis*	Not done
*S. pneumoniae* antigen test from urine	Negative	Positive	Positive	Not done	Positive	Positive	Not done
Chest radiograph findings	Alveolar consolidation, pleural effusion	Alveolar consolidation	Alveolar consolidation	Interstitial infiltrates	Alveolar consolidation, dense	Alveolar consolidation, dense	Alveolar consolidation
Duration of symptoms before referral, days	3	3	4	1	7	1	2
Length of fever in hospital, days	1.5	<1	<1	1.5	<1	<1	<1
Length of hospitalization, days	2	2	2	2	2	1	2
Antibiotic treatment received during hospitalization	Penicillin + azithromycin	Penicillin	Penicillin	Cefuroxime -ceftriaxone	Penicillin	Penicillin	Penicillin—cefuroxime

**Figure 1:**
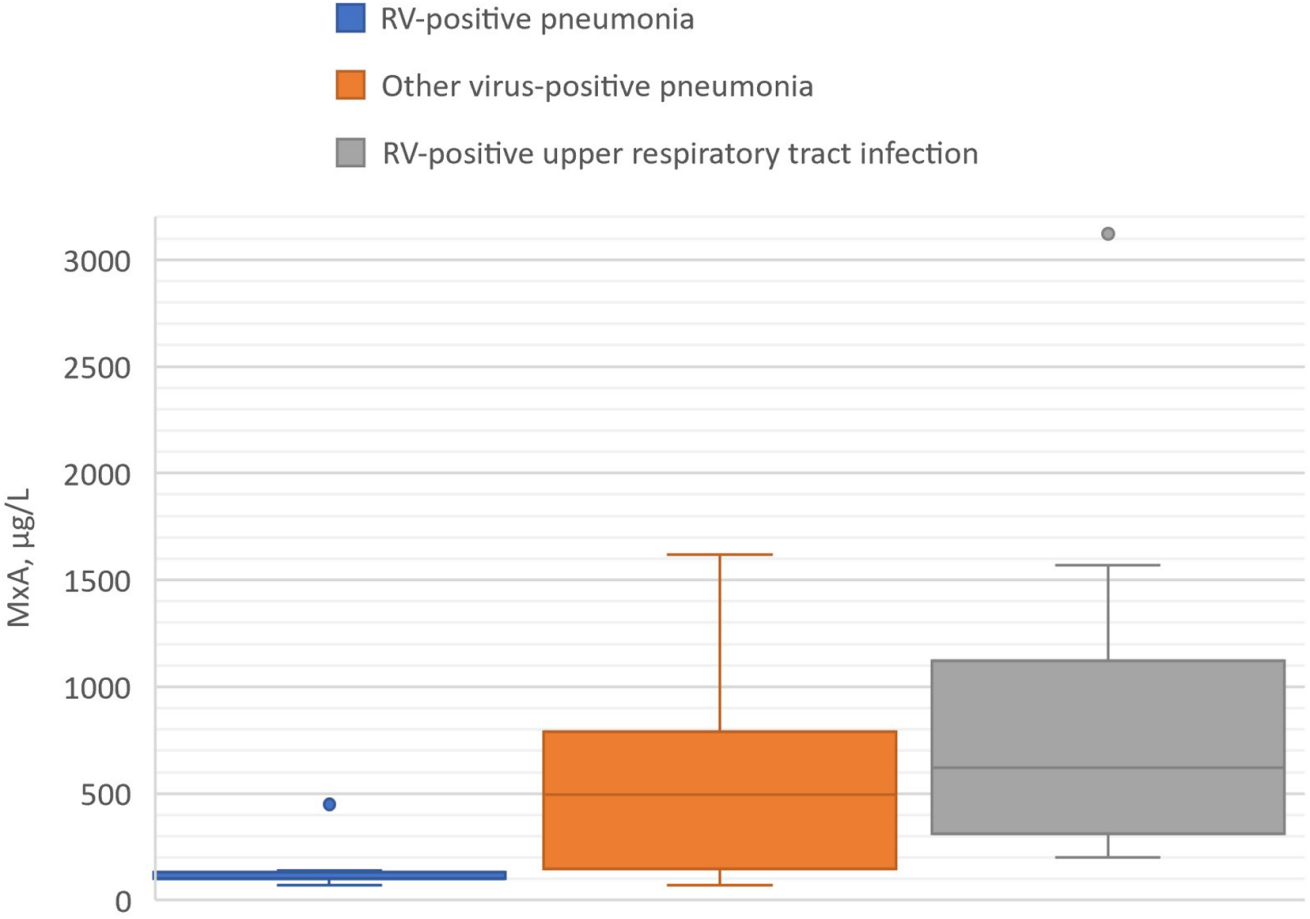
Blood MxA levels in children with RV-positive pneumonia, other virus-positive pneumonia, and RV-positive upper respiratory tract infection (viral coinfections excluded). Boxes show medians and IQRs. Whiskers show the largest and smallest values with the exception of two outlier values exceeding 1.5 times the IQR shown by dots.

Blood MxA concentration was measured from 6 children with RV pneumonia and no other viral findings (median age 1.64 years). It was detectable in all cases but concentrations were low varying from 70 to 450 μg/L (median 100 μg/L). The levels were lower than in children (*n* = 10) with pneumonia positive for another respiratory virus (median 495 μg/L, range 70–1620 μg/L, *p* = 0.034) (Table 2, Figure 1). The MxA levels in children with RV-positive pneumonia were also lower than the levels in children with RV-positive URTI (median 620 μg/L, range 200–3120 μg/L, *p* = 0.011) (Table 3, Figure 1). The median Ct value for RV was lower (23.2) in children with RV pneumonia compared to children with RV URTI (28.7), suggesting higher viral load in children with pneumonia, but the difference was not significant (Table 3).

**Table 2:** The clinical characteristics of pneumonia in RV-positive and other virus-positive children (viral coinfections were not included).

	RV positive, *n* = 6	Another virus positive, *n* = 10^*^	*P*-value
Males	5 (83)	5 (50)	0.307
Age, year	1.64 (1.06–3.77)	3.26 (2.49–7.09)	0.174
**Symptoms**			
Cough	6 (100)	10 (100)	
Fever	6 (100)	9 (90)	1.000
Rhinitis	6 (100)	8 (80)	0.500
Vomiting	5 (83)	6 (60)	0.588
Nasal congestion	5 (83)	6 (60)	0.588
Dyspnea	3 (50)	3 (30)	0.607
Hoarse voice	3 (50)	8 (80)	0.299
**Laboratory findings**			
Highest WBC count, ×10^9^/L	17.9 (13.4–20.6)	13.7 (10.0–27.0)	0.724
Highest CRP, mg/L	294 (239–335)	223 (112–284)	0.084
Highest PCT, µg/L	6.61 (4.74–16.69)	2.29 (0.61–6.85)	0.058
MxA, µg/L	100 (93–218)	495 (108–855)	0.034
**Chest radiograph findings**			
Alveolar consolidation	5 (83)	6 (60)	0.588
Interstitial infiltrates	1 (17)	4 (40)	0.588
**Other characteristics**			
Antibiotic treatment received before referral	1 (17)	3 (30)	1.000
Antibiotic treatment received during hospitalization	6 (100)	10 (100)	
Duration of symptoms before referral, days	2.5 (1.0–4.8)	4.0 (3.0–10.0)	0.150
Length of hospitalization, days	2.0 (1.8–2.0)	1.0 (1.0–2.0)	0.041

Values are *n* (%) or median (IQR). For categorical data, comparisons were performed by use of Fisher's exact test, and, for continuous data, comparisons were performed by the *t* test.

* Respiratory syncytial virus *n* = 3, human metapneumovirus *n* = 2, adenovirus *n* = 1, coronavirus *n* = 1, enterovirus *n* = 1, human bocavirus *n* = 1, influenza A virus *n* = 1.

**Table 3:** Comparison of children with RV-positive pneumonia and children with RV-positive upper respiratory tract infection (URTI).

	RV-positive pneumonia, *n* = 6	RV-positive URTI, *n* = 13	*P*-value
Age, year	1.64 (1.06–3.77)	0.79 (0.64–1.09)	0.095
Cycle threshold (Ct) value of RV	23.2 (21.5–26.1)	28.7 (25.8–31.7)	0.135
MxA, µg/L	100 (93–218)	620 (310–1120)	0.011

Values are median (IQR). Comparisons were performed by the *t* test.

The clinical characteristics of pneumonia were similar in RV-positive and other virus-positive children (Table 2). In all 7 RV-positive children with pneumonia the biomarkers for bacterial infection were markedly increased: the median (range) of highest values for WBC was 18.0 (10.3–25.7) × 10^9^/L, for CRP 290 (190–345) mg/L, and for PCT 6.61 (1.99–25.73) μg/L. In children with pneumonia positive for another virus the levels of the markers of bacterial infection were also high (Table 2). One RV-positive child had a blood culture positive for *S. pneumoniae*. Chest radiographs revealed alveolar changes in 6 of 7 children with RV pneumonia and in 6 of those 10 with pneumonia positive for another virus. All were treated with antibiotics with rapid recovery (Table 2).

## 4. Discussion

We found evidence of viral infection in 71% of children hospitalized for CAP. This is in line with previous studies which have reported viruses as causative agents of CAP from 62 to 81% of the cases (8–11). RV was detected in 29% of the children in this study. This observation is in agreement with earlier studies in which RV was detected in 23%–30% of children with CAP (8–11).

RV-positive children had detectable blood levels of MxA protein. MxA levels were, however, significantly lower than in those with other virus pneumonias and those with RV-positive URTI. Except one case with a high MxA, the levels were similar to those reported in asymptomatic virus-negative children (12). Young age can contribute to an elevated MxA level, but not as much as was the difference of MxA between our RV-positive pneumonia patients and the comparison group of RV-positive URTI. Low MxA could suggest that RV infection occurred before the onset of pneumonia and the interferon response to virus infection had diminished before patient enrollment. Contrary to this suggestion, Ct values of RV were low (<27), indicating high viral loads and an acute phase of RV infection. In viral-bacterial pneumonia, the bacterial co-infection may dominate the inflammation cascade and downregulate MxA expression. Another possibility is that a relative interferon system deficiency would have resulted in uncontrolled RV replication and more severe clinical illness, as seen in COVID-19 (14).

The role of RV in the etiology of CAP has been considered to be overestimated. Many studies have reported high detection rates in asymptomatic children. One study comprised 121 CAP cases and 240 healthy controls. There were no significant differences in the RV findings between the cases and controls (23% vs. 27%) (11). However, we think that it has not been well recognized that all respiratory viruses, including RV, commonly cause asymptomatic infections. This is well documented now in children with SARS-CoV-2 infections. Furthermore, RV does not induce prolonged viral shedding after an acute infection and thus does not make unspecific “background noise” in diagnostics as some DNA viruses may do. In this study, all children with RV-induced CAP were RV-negative 2 weeks after it was first detected.

Interestingly, the biomarkers for bacterial infections (WBC, CRP, and PCT) were markedly increased in RV-positive children in our study. Moreover, with one exception children had alveolar changes in chest radiograph strongly supporting bacterial infection. The clinical response to antibiotic treatment was rapid in all children with RV-positive pneumonia. Our observations support the earlier reported view that bacterial coinfections are common in RV pneumonia in children (15).

Viral-bacterial co-infections, particularly RV with *S. pneumoniae*, are common in childhood CAP and possibly associated with a more severe course of illness (8, 9). The seasonality of viral lower respiratory tract infections coincides with both bacteremic and nonbacteremic pneumonia (16), and a temporal association has been shown between increased RV circulation in the community and invasive pneumococcal disease in children younger than 5 years (17). RV-induced severe CAP with or without bacterial coinfection has also been reported in adults (18). Interestingly, RV impairs immune responses to bacterial products and phagocytosis of bacteria in human macrophages (19, 20). Other mechanisms, such as increased permeability of airway epithelium and stimulated adhesion of *S. pneumoniae* to airway epithelial cells, are also involved in the synergism between RV and bacteria (21, 22).

There are limitations in our study. In this pilot study the number of children with RV-positive CAP was low, and therefore, the results should be interpreted with caution. The comparison group of children with RV-positive URTI consisted of younger children than the RV pneumonia group, though without statistically significant age difference. Like most previous studies on RV pneumonia, our study was a PCR-based study. Detection of RVs also by culture, which reflects virus replication, would have strengthened our conclusions. Collection of samples by bronchoalveolar lavage, which is not routinely done, would have supported the role of RV as a cause of pneumonia (23). It should be noted that bacterial culture from nasopharyngeal swab and *S. pneumoniae* antigen test from urine can detect bacterial colonization and are not diagnostic methods for pneumonia in children.

In conclusion, we commonly detected RV in children hospitalized with CAP. Our observations, including a high viral load followed by rapid clearance of RV, suggest that RV may have a pathogenic role in CAP, but bacterial coinfections are common. Low MxA levels in children with RV-positive CAP need further studies but suggest an aberrant interferon response to RV in children with viral-bacterial pneumonia.

## Data Availability

The original contributions presented in the study are included in the article/Supplementary Material, further inquiries can be directed to the corresponding author/s.
